# Effectiveness and satisfaction with virtual and donor dissections: A randomized controlled trial

**DOI:** 10.1038/s41598-024-66292-7

**Published:** 2024-07-16

**Authors:** Young Hyun Yun, Hyeok Yi Kwon, Su Kyoung Jeon, Yu Mi Jon, Min Jung Park, Dong Hoon Shin, Hyung Jin Choi

**Affiliations:** 1 Department of Anatomy and Cell Biology, Seoul National University College of Medicine, Seoul, Republic of Korea; 2 Department of Neurosurgery, Seoul National University Hospital, Seoul, Republic of Korea; 3 Department of Biomedical Sciences, Seoul National University College of Medicine, Seoul, Republic of Korea

**Keywords:** Scenario-based virtual content, Virtual dissection, Head-mounted display, Life-sized touchscreen, Tablet, Heart anatomy, Neuroanatomy, Anatomy, Cardiology, Neurology

## Abstract

In recent years, human anatomy education has faced challenges with traditional donor dissection, leading to the emergence of virtual dissection as an alternative. This study aims to investigate the academic performance and satisfaction of medical students by comparing the virtual and donor dissections. An open-labeled crossover randomized controlled trial was conducted with 154 first-year medical students in Human Anatomy and Neuroanatomy laboratories, which were divided into three classes. Students were randomly assigned to either the virtual (virtual dissection followed by donor dissection) or donor (donor dissection followed by virtual dissection) groups in each class. A curriculum, incorporating head-mounted displays (HMDs), a life-sized touchscreen, and tablets, was developed. Data was evaluated through quizzes and surveys. In the Human Anatomy laboratory, each class of the donor group conducted heart extraction, dissection and observation. In observation class, the virtual group had a significantly higher mean quiz score than the donor group (*p* < 0.05). Compared to the donor, satisfaction was significantly higher for the HMD (understanding of concept and immersion), life-size touchscreen (esthetics, understanding of the concept, and spatial ability), and tablet (esthetics, understanding of the concept, spatial ability, and continuous use intention). In the Neuroanatomy laboratory, the virtual group showed significantly higher mean quiz scores than the donor group (*p* < 0.05), and tablet showed a significantly higher satisfaction than donor in terms of esthetics, understanding of the concept, and spatial ability. These results suggest that virtual dissection has the potential to supplement or replace donor dissection in anatomy education. This study is innovative in that it successfully delivered scenario-based virtual content and validated the efficacy in academic performance and satisfaction when using virtual devices compared to donor.

Trial registration: This research has been registered in the Clinical Research Information Service (CRIS, https://cris.nih.go.kr/cris/search/detailSearch.do?search_lang=E&focus=reset_12&search_page=L&pageSize=10&page=undefined&seq=26002&status=5&seq_group=26002) with registration number "KCT0009075" and registration date "27/12/2023".

## Introduction

Traditional human anatomy education has historically relied on body donors for studying the structure of human body. However, emerging issues such as donor shortages, high costs, formaldehyde exposure, ethical concerns, and challenges posed by pandemics are now making it increasingly difficult to use body donors^1,2^. Consequently, universities have reduced anatomy laboratory hours and even eliminated body donor-based instruction from some curricula^3,4^. In response to these challenges, virtual dissection has emerged as a viable alternative to traditional methods in anatomy education^5^.

Virtual dissection is an innovative method that employs digital devices and advanced three-dimensional (3D) technology to simulate anatomical models in a virtual environment^6^. Accessible through devices such as head-mounted displays (HMDs) and conventional touchscreens or tablets, this method offers unique opportunities for exploring anatomical structures in ways not possible with traditional methods^7^. During virtual dissection, users can manipulate virtual anatomical models, adjust transparency, and apply colorization to better understand the intricate structures of the human body^8^.

The adoption of various devices is transforming anatomy education by providing diverse learning experiences. Virtual Reality HMDs enable immersion in interactive 3D environments, enhancing technical skills, anatomy teaching, surgical training, and cardiopulmonary resuscitation^9–12^. Additionally, mobile learning devices, such as smartphones and tablets, have become popular due to their versatility and multifunctionality. Most medical students own these devices, and this trend is increasing^13–15^. Virtual dissection tables are also increasingly being adopted by many leading medical schools and institutions worldwide. These are an all-in-one touch-interactive display system and come equipped with a built-in digital imaging and communications in medicine viewer for displaying computed tomography and magnetic resonance imaging scans^16^. Numerous studies supporting the specific use of visualization tables and other 3D visualization technologies have contributed to this trend^17–19^.

3D anatomy software is increasingly used in anatomy education as smartphone and tablet applications gain popularity^20^. Novel commercial anatomy applications enable students to explore anatomical structures through 3D models, providing flexibility in viewing and manipulation^21^. As highlighted by Park group paper^22^, most students find 3D anatomy software more effective than traditional two-dimensional atlases^23^. However, achieving effective virtual anatomy education goes beyond just transitioning from body donors to virtual methods; it requires anatomy content scenarios. Specialists are needed to supervise the design of virtual anatomical content scenarios^24^. Digital learning scenarios guide learners in a digital environment, requiring careful planning that considers target learners, goals, objectives, intended outcomes, and contexts^25^.

The complexity of structure such as human heart and brain highlight the necessity for effective educational methods. In donor dissection, medical students often encounter difficulties in understanding the anatomical structure and positional relationships of the heart, primarily due to its location within the mediastinum and encased by the thorax^26,27^. Similarly, neuroanatomy is another challenging subject for many novice students^28–30^. This requires students to thoroughly understand numerous neuroanatomical structures, vessels, and nerves and their complex spatial relationships^31^. Therefore, it is imperative for educational approaches to provide effective methods for students to grasp these intricate anatomical structures.

While several studies have explored the academic performance and satisfaction of medical students using virtual dissection, the full substitution of conventional anatomy education with virtual approaches remains unrealized. These studies have primarily focused on comparing donor dissections with virtual dissections limited to specific devices and were conducted without anatomy content scenarios. Therefore, there is a necessity to develop anatomy content scenarios and compare them across various devices with donor dissections.

The goal of college courses is to produce learning outcomes, which are of considerable interest to students, professors, and researchers in higher education^32^. Student performance serves as a measure of teaching effectiveness^33^. Therefore, comprehending students' learning style preferences holds immense potential across various educational contexts. It ranges from categorizing students' learning preferences to identifying potential learning challenges early on, enabling educators to select suitable teaching methodologies accordingly.

Student satisfaction in the classroom is an inherently desirable goal and a significant benefit of effective teaching^34^. When students are satisfied with their learning experiences, they are more likely to be engaged, motivated, and successful in their studies^35^. With different learning modalities, student satisfaction becomes even more critical. Understanding how each modality impacts student satisfaction is essential for optimizing the educational experience. Overall, understanding student satisfaction are crucial components in evaluating the efficacy of different learning approaches and technologies, ultimately contributing to the success of educational programs and platforms.

This study hypothesized the conceptual framework that there will be differences in academic performance between virtual and donor dissections in different dissection situations and that virtual dissection has the potential to supplement or replace traditional donor dissection. Additionally, the study further hypothesized that there would be differences in satisfaction levels between various virtual dissection methods, including HMDs, life-size touchscreen, and tablets, and traditional methods.

The aim of this study was to develop curriculum and scenario-based virtual content and investigate students’ academic performance and satisfaction by comparing the virtual and donor dissections. Our three specific research questions guided this study: (1) Are there differences in student academic performance when doing virtual and traditional donor dissections?; (2) Are there differences in students’ satisfaction when doing virtual and traditional donor dissections?; and (3) Can virtual dissection supplement or replace traditional donor dissection?

## Methods

### Sample size

We assumed that 62 ± 11 versus 68 ± 11 in the mean quiz scores between the donor and virtual groups would indicate a meaningful effect. To detect this difference in means with a significance level of 95% and a power of 0.90, we estimated that a total sample size of 142 students would be necessary.

### Participants

The study included 154 first-year medical students who voluntarily enrolled in Human Anatomy and Neuroanatomy courses and were at least 19 years old on the date of study entry at the Seoul National University College of Medicine, Seoul, Republic of Korea, in 2021. All participants were recruited in person before the courses began, and no additional grades are provided as compensation for participation in the study.

### Curriculum development

Based on the Analysis, Design, Development, Implementation, and Evaluation (ADDIE) model^36^, we developed and applied an anatomical education curriculum using digital technologies and evaluated its effectiveness. Supplementary Table S1 presents the curriculum-development process used in this study.

### Study design

This open-labeled crossover randomized controlled trial involved two intervention arms: virtual and donor groups. Students were randomly assigned, using a lottery method, to undergo either donor dissection followed by virtual dissection or vice versa. Each laboratory session lasted for 2 h, with both the virtual and donor dissections taking place within a 50–54-min timeframe. Following the first dissection, students completed a 3–6-min Quiz 1 (Q1, primary outcome). Students also completed a 3–6-min Quiz 2 (Q2, secondary outcome) and a 6–8-min survey at the end of the laboratory sessions.

### Study design of the “Human Anatomy” laboratory

**Figure 1:**
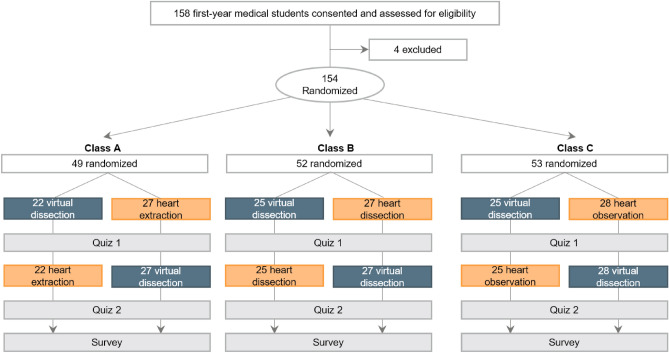
Study flow diagram of the Human Anatomy laboratory. The virtual dissection laboratory includes head-mounted displays, life-sized touchscreens, and tablets.

A flow diagram of the Human Anatomy laboratory is shown in Fig. 1. Due to constraints imposed by the coronavirus disease 2019 (COVID-19), which required smaller group sizes to comply with safety protocols, the 154 students were randomly divided into three classes: class A (n = 49), class B (n = 52), and class C (n = 53). In each class, the students were randomly assigned to either virtual group (virtual dissection → donor dissection) or the donor group (donor dissection → virtual dissection). Each group comprised only 21–28 students. Overall, 72 students were in the virtual group and 82 in the donor group. In the donor dissection, three situations were presented, contributing to different student experiences: heart extraction (class A), dissection (class B), and observation (class C). Supplementary Figure S1 illustrates the schedule of the virtual dissection using HMD, life-sized touchscreens, and tablets. The three virtual devices were used in the virtual dissection lab for 54 min (Supplementary Figure S1a). Every 18 min, three teams with nine students rotated and had the opportunity to experience the three devices. Each student spent 6 min using the HMD and the remaining 12 min observing (Supplementary Figure S1b). Nine students used the life-sized touchscreen (Supplementary Figure S1c), while a tablet was assigned to each student (Supplementary Figure S1d).

### Study design of the “Neuroanatomy” laboratory

**Figure 2:**
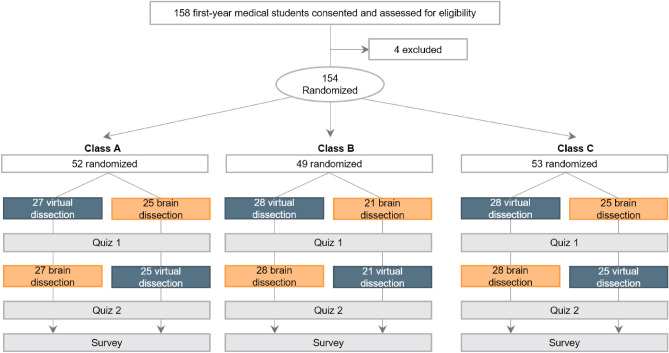
Study flow diagram of the Neuroanatomy laboratory. The virtual dissection laboratory uses head-mounted displays, life-sized touchscreens, and tablets.

Figure 2 shows the flow diagram of the neuroanatomy laboratory. Due to COVID-19, all 154 students were randomly assigned to three classes: class A (n = 52), class B (n = 49), and class C (n = 53). Students in each class were randomly divided into two groups: a virtual group (virtual dissection → donor dissection) and a donor group (donor dissection → virtual dissection). All classes conducted donor dissections under the same conditions. Additionally, for virtual dissection, students used only tablets.

### Learning resources for donor and virtual dissections

The Human Anatomy 7^th^ edition guidelines (Korea Medical Book Publishing Company, Seoul, Korea) and Seven Days of Neuroanatomy Lab: Manual and Atlas (Panmun Education, Seoul, Korea) were used as the primary textbooks for donor dissection. Three types of devices were used in this study: Anatomage Table™ (Anatomage, Inc., Santa Clara, California, USA), iPads® (Apple Inc., Cupertino, California, USA), MDBox™ (Medical IP Co., Ltd., Seoul, Republic of Korea) with Oculus Quest 2 HMDs (Meta Platforms, Inc., Menlo Park, California, USA) and two touches. Additionally, three anatomy software programs were used: Anatomage™ (Anatomage, Inc., Santa Clara, California, USA), Complete Anatomy® (3D4Medical, San Diego, California, USA) and MDBOX™ (version 1) (Medical IP Co., Ltd., Seoul, Republic of Korea). The devices were selected for this study based on their availability and accessibility, as they were located within the anatomy laboratory. The Anatomage™ and MDBOX™ devices were equipped with their own software, while access to Complete Anatomy® was through a school subscription.

### Donor dissection in “Human Anatomy” and “Neuroanatomy” laboratories

Tutors prepared the donor dissection in advance using primary textbooks and conducted the dissection. They also provided detailed explanations and guidance to students during the dissection sessions.

### Content scenario for virtual dissection and donor dissection in “Human Anatomy” laboratory

The virtual content was developed according to the practical sequence outlined in the primary textbook. The contents are organized in a specific order, starting with the coronary artery, followed by the chamber, valve, and conducting system. The tutors prepared virtual content scenarios in advance using an HMD (Supplementary Figure S2), a life-sized touchscreen (Supplementary Figure S3), and a tablet (Supplementary Figure S4). The specific contents explained by the tutor under each topic can be found in Appendix S1. The heart content was stored in the library and accessible through preset tabs in HMD-based (Supplementary Video 1), life-sized touchscreen-based (Supplementary Video 2), and tablet-based laboratories (Supplementary Video 3).

### Content scenario for virtual dissection of “Neuroanatomy” laboratory

The contents covered various diencephalon and telencephalon structures and structures associated with the third and lateral ventricles. The tutors prepared scenarios for the content in advance using a tablet (Fig. S5). Detailed contents explained by the tutor for each topic can be found in Appendix S2. The contents were stored in the library and accessible through preset tabs in the tablet-based laboratories (Supplementary Video 4).

### Quiz and survey

The first quiz aimed to assess students' understanding after experiencing either donor or virtual dissection, focusing on the differences in scores between these two learning modalities. The main outcome of interest was to observe any variations in scores based on the type of dissection experienced. In contrast, the second quiz was administered after students had experienced both donor and virtual anatomy dissection. The aim was to assess whether there were differences in achievement levels based on the sequence of exposure to donor and virtual dissections. Both quizzes, prepared by the faculty, consisted of 7 image-based multiple-choice questions each, and each quiz lasted for 3–6 min. The quizzes were conducted under exam conditions.

The survey questionnaire was chosen to evaluate the educational satisfaction of virtual dissection compared to donor dissection without consideration of gender distribution. An online Google Forms questionnaire asked students about their demographics (gender and age) and satisfaction with the virtual and donor dissection. Students were asked to evaluate satisfaction with the donor, HMD, life-sized touchscreen, and tablet using 5-point Likert-type scale score (1 = very unsatisfied and 5 = very satisfied) on 6 categories with 3 subscales; esthetics (utility, design, and vividness), understanding of the concept (labeling, desired angles, and comprehension), reality (anatomical structures, environment, and real world), spatial ability (facilitated observation, intuitive understanding, and spatial perception), immersion (desire to continue, sense of being in another space, and forget about daily life), and continuance usage intention (re-experience, repetition, and experience in different structures). Appendix S3 presents the questions for each subscale within the categories.

### Statistical analysis

All statistical analyses were performed using SPSS version 26 (IBM Corp., Armonk, NY, USA). We used the 2-tailed Student’s t-test to assess the differences in quiz scores between the virtual and donor groups and considered *p* < 0.05 statistically significant. In addition, we used one-way ANOVA and Scheffe’s post hoc tests to identify differences between donor, HMD, life-sized touchscreen, and tablet. For the results not following a normal distribution, we applied nonparametric Kruskal–Wallis H test with Bonferroni post hoc tests. Additionally, we used the Mann–Whitney U test to compare the difference in satisfaction levels between the donor and tablet. The Cronbach's Alpha overall reliability coefficient for the questionnaire in the Human Anatomy laboratory was calculated as 0.917, and for Neuroanatomy, it was 0.914.

### Ethical approval

This study was approved by the Institutional Review Board of Seoul National University Hospital (IRB number: C-2103-066-1203), and all methods were performed in accordance with relevant guidelines and regulations. Students were voluntarily participated in this study, and informed consent was obtained from each student before the study. Students not participating in this study were also provided equal opportunities for virtual and donor dissection laboratories. In addition, informed consent for body donation was obtained from the donor or their next of kin.

## Results

### Academic achievement

**Figure 3:**
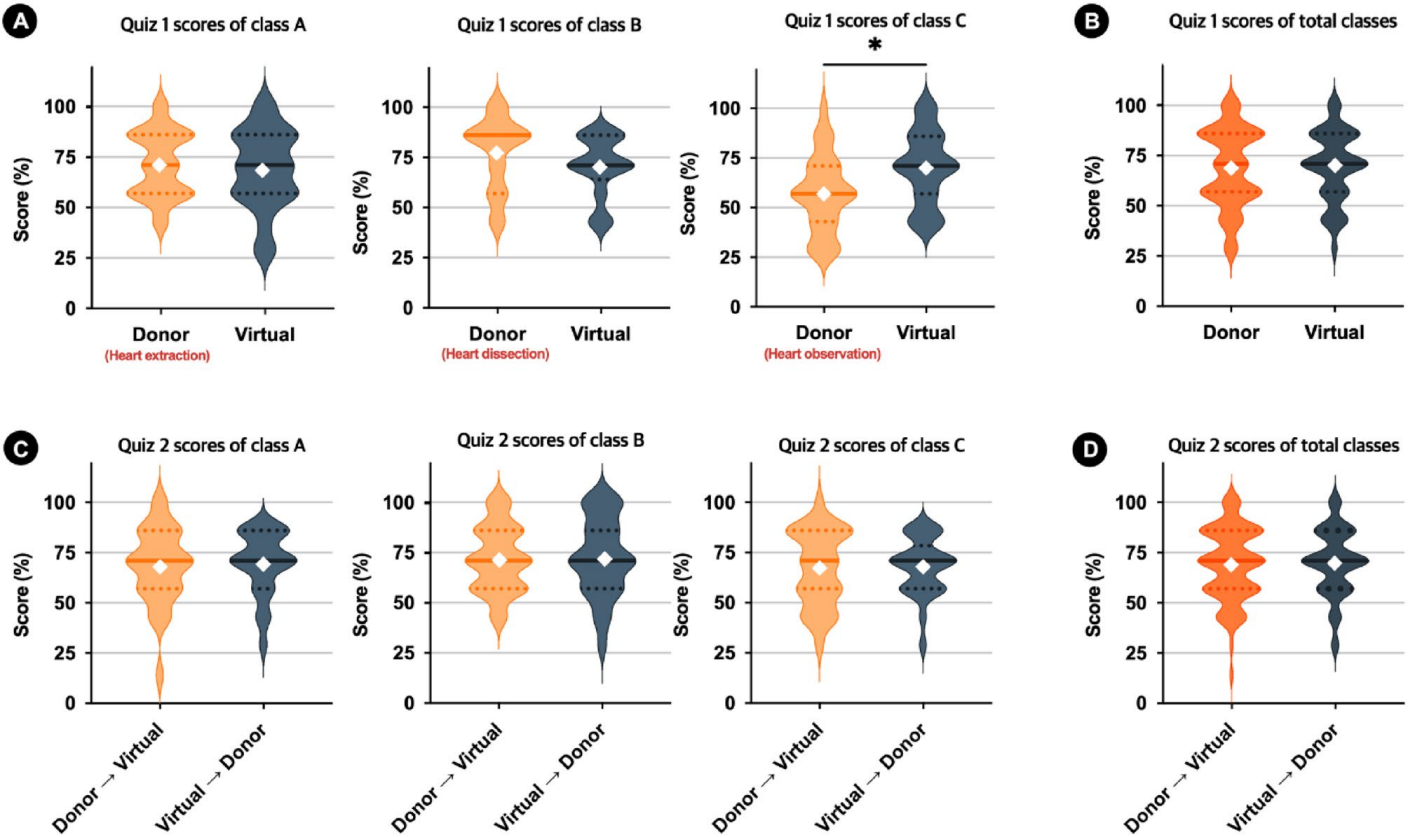
Comparison of Quiz 1 (Q1) and Quiz 2 (Q2) scores between the virtual and donor groups in the Human Anatomy laboratory. Students’ academic performance in Q1 and Q2 was assessed using a t-test. (**A**) Violin plots showing the distribution of Q1 scores of classes A, B, and C. (**B**) Violin plots of Q1 scores of all classes. (**C**) Violin plots showing the distribution of Q2 scores of classes A, B, and C. (**D**) Violin plots of Q2 scores of all classes. The dotted line ranges from lower to upper quartiles. The solid line represents median values. White diamonds represent mean. *p < 0.05.

In the Human Anatomy laboratory, Fig. 3 shows the results of Q1 and Q2 mean scores for each three classes (A, B, and C) and the total mean score across all classes. For Q1, in class C where students only observed the heart, the virtual group had a significantly higher mean score than the donor group (*p* < 0.05), but there were no significant differences observed in classes A with heart extraction and B with dissection (Fig. 3A). When all three classes were combined, the mean score of Q1 for the virtual group was slightly higher than that of the donor group; however, the difference was not statistically significant (Fig. 3B). For Q2, there were no significant differences between the two groups within each class (Fig. 3C) or when all three classes were combined (Fig. 3D).

**Figure 4:**
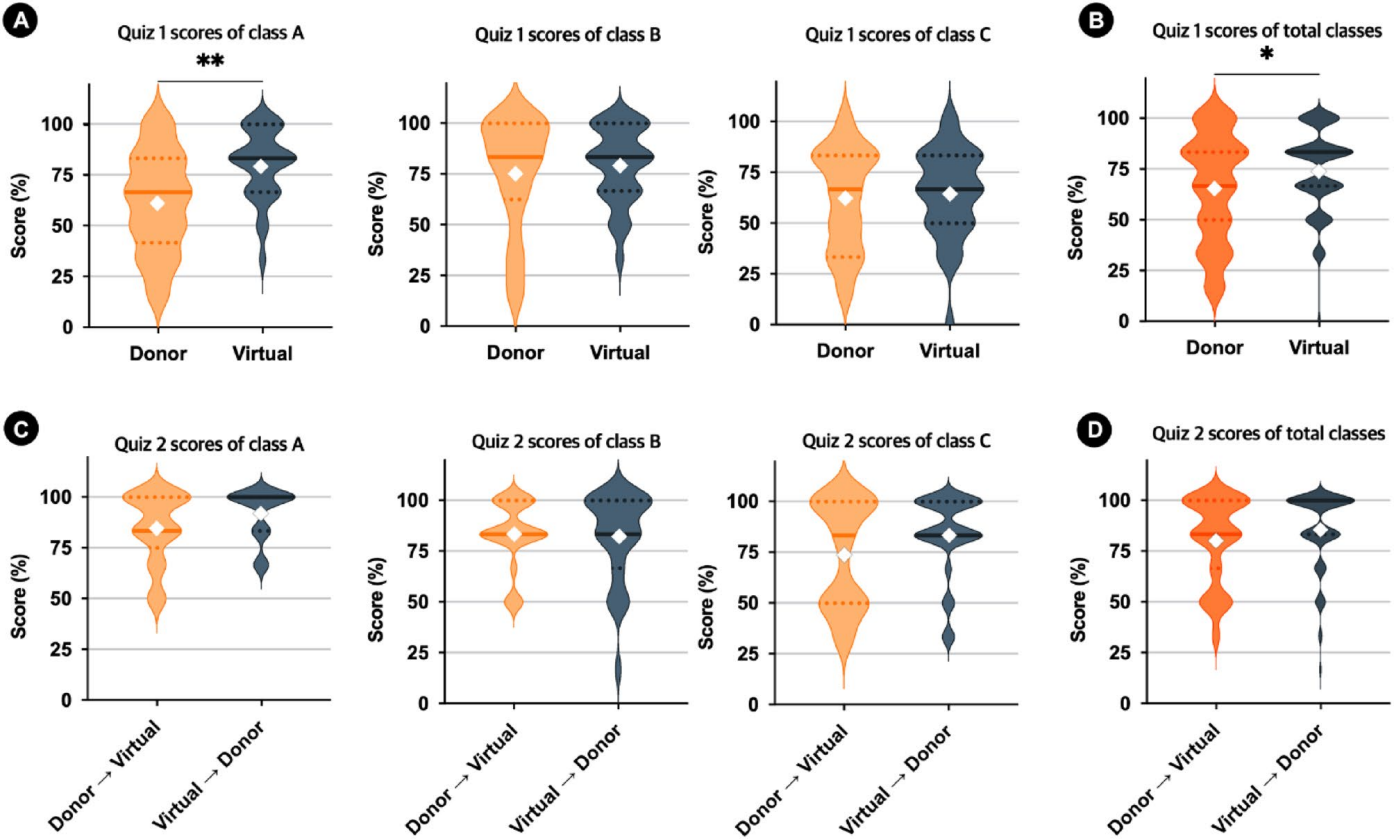
Comparison of Q1 and Q2 scores between the virtual and donor groups in the Neuroanatomy laboratory. Students’ academic performance in Q1 and Q2 was assessed using a t-test. (**A**) Violin plots of Q1 scores of classes A, B, and C. (**B**) Violin plots of Q1 scores of all classes. (**C**) Violin plots Quiz 2 scores of classes A, B, and C. (**D**) Violin plots of Q2 scores of all classes. The dotted line ranges from lower to upper quartiles. The solid line represents median values. White diamonds represent means. *p < 0.05; **p < 0.01.

The virtual and donor groups in the Neuroanatomy laboratory had identical laboratory conditions for all the classes. Figure 4 represents the results of Q1 and Q2 scores. The Q1 mean score was statistically significant (*p* < 0.01) in class A, indicating that the virtual group performed at a significantly higher level than the donor group (Fig. 4A). Although the virtual group in classes B and C had higher mean scores than the donor group, no significant differences were observed. As shown in Fig. 4B, the Q1 mean score of all classes was significantly higher in the virtual group than in the donor group (*p* < 0.05). For Q2, there were no statistically significant differences between the two groups within each class (Fig. 4C), and when all three classes were combined (Fig. 4D).

### Demographic and other characteristics of medical students

In Human Anatomy laboratory, 154 first-year medical students were attended this study (Table 1). The mean (SD) age of students was 20.8 (1.2) years, with a majority being male (69.5%) and the remaining female (30.5%). While 54.5% students owned a personal tablet and 6.5% used a shared family tablet, 39.0% reported not having one. Among the 92 students who owned a tablet, most (76.1%) used it for educational purposes, while 4.3% used it for gaming and 19.6% for watching videos or movies. Over half of the students (59.7%) reported never using VR. The percentage of students using it 2–3 times a week, once a week, or once a day was minimal (0.6% each). Among the 62 students experienced in using VR technology, the majority (87.1%) used it for gaming, while 9.7% used it for watching videos or movies. Only 1.6% participated in both research and educational activities. Among the students who played VR games, 40 (26.0%) played once a year, 7 (4.5%) played once a month, 1 (0.6%) played 2–3 times a week, 4 (1.9%) played once a week, and 1 (0.6%) played once a day. Additionally, 104 students (67.5%) did not use graphic design software.

**Table 1:** Demographic and other characteristics of Human Anatomy survey respondents.

Characteristic	Student, No. (%) First-year medical students (n = 154)^a^
Age, mean (SD)	20.8 (1.2)
Gender	
Male	107 (69.5)
Female	47 (30.5)
Do you have a tablet?	
Personal tablet	84 (54.5)
Shared family tablet	10 (6.5)
Purpose of tablet usage (n = 92)*	
Education	70 (76.1)
Game	4 (4.3)
Video or movie	18 (19.6)
No	60 (39.0)
Frequency of VR usage	
Once a day	1 (0.6)
Once a week	1 (0.6)
2–3 times a week	1 (0.6)
Once a month	4 (2.6)
Once a year	55 (35.7)
What was your experience with VR? (n = 62)*	
Game	54 (87.1)
Education	1 (1.6)
Video or movie	6 (9.7)
Other^b^	1 (1.6)
Never	92 (59.7)
Frequency of VR games based on a first-person perspective	
Once a day	1 (0.6)
Once a week	4 (1.9)
2–3 times a week	1 (0.6)
Once a month	7 (4.5)
Once a year	40 (26.0)
Never	101 (65.6)
Frequency of graphic design software usage	
Once a day	3 (1.9)
Once a week	3 (1.9)
2–3 times a week	3 (1.9)
Once a month	18 (11.7)
Once a year	23 (14.9)
Never	104 (67.5)

*VR* virtual reality.

^a^Data are presented as the number (percentage) of survey respondents unless otherwise indicated.

^b^Other studies included only research participants.

*Only one subgroup of students.

In Neuroanatomy laboratory, 154 out of 158 total students attended. As detailed in Table 2, the mean (SD) age of students was 21.9 (9.4%). The majority were male (70.8%), with only 29.2% being female Students reported owning either a personal tablet (93.5%) or a family tablet (1.3%), while 8% stated not having a tablet. Among the 146 students who used a tablet, more than half (59%) utilized it once a month. Additionally, students (70.1%) bought virtual anatomy applications, mainly using Complete Anatomy (56.5%), followed by atlas (9.1%), anatomical learning (3.9%), and 3D anatomy (0.6%). Among the 108 students who purchased virtual anatomy applications, 11.7% used them once a day, 12.3% once a week, 32.5% 2–6 times a week, 9.7% 2–3 times a month, and 3.9% once a month.

**Table 2:** Demographic and other characteristics of Neuroanatomy survey respondents.

Characteristic	Student, No. (%) First year medical students (n = 154)^a^
Age, mean (SD)	21.9 (9.4)
Gender	
Male	109 (70.8)
Female	45 (29.2)
Do you have a tablet?	
Personal tablet	144 (93.5)
Shared family tablet	2 (1.3)
Frequency of tablet usage (n = 146)*	
Once a day	17 (11%)
Once a week	5 (3.2%)
2–6 times a week	35 (22.7%)
2–3 times a month	6 (3.9%)
Once a month	91(59%)
No	8 (5.2)
Did you buy virtual anatomy applications?	
Yes	108 (70.1%)
What is the name of the virtual anatomy application? (n = 108)*	
Complete Anatomy	87 (56.5%)
Atlas	14 (9.1%)
Anatomy Learning	6 (3.9%)
3D Anatomy	1 (0.6%)
Frequency of virtual anatomy applications usage (n = 108)*	
Once a day	18 (11.7%)
Once a week	19 (12.3%)
2–6 times a week	50 (32.5%)
2–3 times a month	15 (9.7%)
Once a month	6 (3.9%)
No	46 (29.9%)

^a^Data are presented as the number (percentage) of survey respondents unless otherwise indicated.

*Only one subgroup of students.

### Student satisfaction

**Figure 5:**
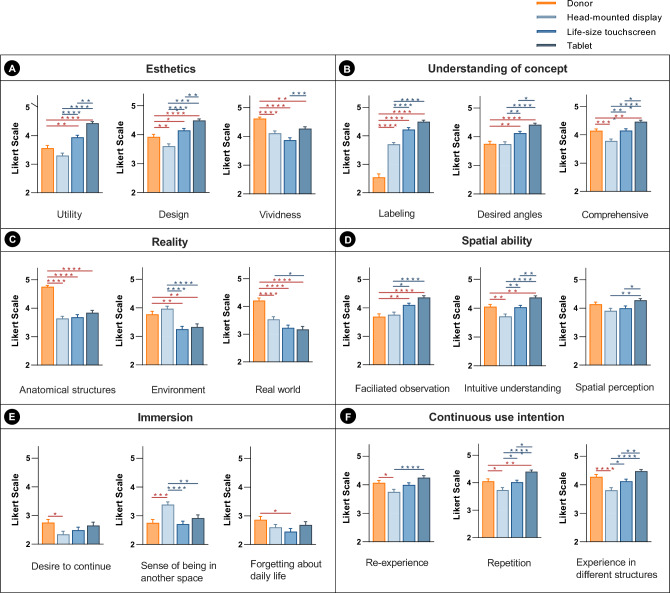
Comparison satisfaction with the three virtual devices and donor on 6 categories with 3 subscales in the Human Anatomy laboratory. Satisfaction was ranked on a 5-point Likert-type scale (1 = very unsatisfied and 5 = very satisfied). The categories are as follows: (**A**) esthetics, (**B**) understanding of the concept, (**C**) reality, (**D**) spatial ability, (**E**) immersion, and (**F**) continuous use intention. The red pairwise comparison on the bar indicates a significant difference between the virtual devices and donor. The blue pairwise comparison in the bar represents a statistically significant difference between virtual devices. *p < 0.05; **p < 0.01; ***p < 0.001; ****p < 0.0001.

In Human Anatomy laboratory, students’ satisfaction in responding to the donor for dissection and three devices (i.e., HMD, life-size touchscreen, and tablet) for virtual dissection was investigated on a 5-point Likert scale (Fig. 5). Regarding utility and design, the life-size touchscreen and tablet showed significantly higher satisfaction compared to the donor (Fig. 5A). Among the three virtual devices, satisfaction with tablet was the highest. However, concerning vividness, the donor showed significantly higher satisfaction than the virtual devices. After nonparametric statistics, utility was significant in all comparisons except for tablet and HMD, and tablet and life-size touchscreen. For design, all comparisons were significant except for tablet and life-size touchscreen. In terms of vividness, only comparison between HMD and life-size touchscreen, and HMD and donor were not significant.

With regard to labeling, the three virtual devices showed significantly higher satisfaction than the donor (Fig. 5B). Additionally, regarding desired angle, the life-size touchscreen and tablet revealed significantly higher satisfaction than the donor. In terms of comprehensiveness, the tablet showed higher satisfaction levels than the donor. However, after nonparametric statistics, labeling showed significance in all comparisons except for HMD and donor. In terms of desired angles, all comparisons were significant except for tablet and life-size touchscreen, and tablet and HMD. Regarding vividness, only comparison between life-size touchscreen and tablet was not significant.

Regarding the anatomical structures and real world, the donor showed significantly higher satisfaction than the three virtual devices (Fig. 5C). Similarly, satisfaction with the environment was significantly higher for donor than life-sized touchscreens and tablets. However, after nonparametric statistics, significance in terms of anatomical structures was observed only between tablet and HMD, tablet and life-size touchscreen, and tablet and donor. For the environment, significance was absent only between HMD and donor, and HMD and tablet. In the real world, significance was found only between tablet and donor, tablet and life-size touchscreen, and tablet and HMD.

In terms of facilitated observation, life-size touchscreen and tablet showed significantly higher satisfaction than the donor (Fig. 5D). Additionally, regarding intuitive understanding, the tablet showed significantly higher satisfaction than the donor, but the satisfaction of donor was higher than HMD. In terms of spatial perception, the three virtual devices showed no significant differences compared to the donor. However, after nonparametric statistics, significant differences in facilitated observation were only observed between the donor and the three virtual devices. Regarding intuitive understanding, significance was found between HMD and life-size touchscreen, as well as donor and three devices. In terms of spatial perception, the donor showed significant differences only with the HMD.

Regarding the sense of being in another space, the HMD showed significantly higher satisfaction than the donor, life-sized touchscreen, and tablet (Fig. 5E). However, regarding the desire to continue and forget about daily life, the donor showed significantly higher satisfaction than the HMD and life-sized touchscreen, respectively. After nonparametric statistics, significant differences regarding the sense of being in another space were observed only between the HMD and life-size touchscreen, HMD and tablet, and the HMD and donor. No significant difference was found in forgetting about daily life, except between the HMD and tablet, and HMD and donor.

The donor showed significantly higher satisfaction than the HMD in terms of re-experience, repetition and experience in different structures (*p* < 0.0001, respectively) (Fig. 5F). However, after nonparametric statistics, significant differences in re-experience were observed only between the HMD and tablet, and the HMD and donor. In terms of repetition, no significant differences were found only between the HMD and life-size touchscreen, and the life-size touchscreen and tablet. Regarding experiences in different structures, significant differences were observed between the HMD and tablet, the HMD and donor, and the life-size touchscreen and donor.

**Figure 6:**
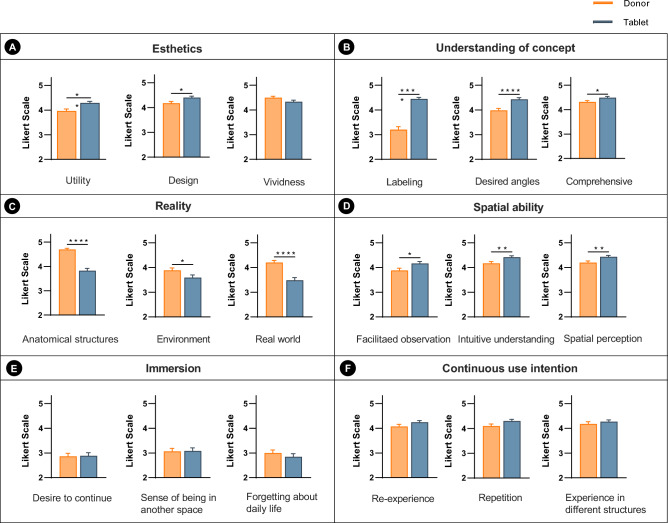
Comparison satisfaction with the tablet and donor on 6 categories with 3 subscales in the Neuroanatomy laboratory. The satisfaction was ranked on a 5-point Likert-type scale (1 = very unsatisfied and 5 = very satisfied). The categories are as follows: (**A**) esthetics, (**B**) understanding of the concept, (**C**) reality, (**D**) spatial ability, (**E**) immersion, and (**F**) continuous use intention. *p < 0.05; **p < 0.01; ***p < 0.001; ****p < 0.0001.

In Neuroanatomy laboratory, student satisfaction with the donor and tablet was assessed on a 5-point Likert scale, as depicted in Fig. 6. Regarding esthetics (Fig. 6A), satisfaction with the utility (*p* < 0.01) and design (*p* < 0.05) of the tablet was significantly higher than donor, but there was no significant difference in vividness. In terms of understanding the concept (Fig. 6B) and spatial ability (Fig. 6D) for the three subscales, satisfaction with the tablet was significantly higher than donor, whereas satisfaction with the tablet was significantly lower than donor in terms of reality (Fig. 6C). According to immersion (Fig. 6E) and continuous usage intention (Fig. 6F), there were no significant differences between the donor and tablet on the three subscales.

## Discussion

The purpose of our study was to provide developed curriculum and scenario-based virtual anatomy content for Human Anatomy and Neuroanatomy laboratories and to investigate whether virtual dissection can supplement or replace donor dissection in terms of students’ academic performance and satisfaction. This study suggests that not only the tools but also tutors, lectures, and the design and pedagogy of the learning process can contribute to students' learning outcomes and satisfaction.

Our study is significant in that we developed a virtual anatomy learning curriculum based on the ADDIE model with careful consideration of pedagogical goals and methods for the learning process, and compared it to traditional anatomy teaching methods. Previous studies have reported the application of the ADDIE model to anatomy education, but there is a paucity of research on detailed digital anatomy curricula. In addition, several meta-analyses have shown no statistical difference in student performance scores when comparing traditional anatomy to other anatomy resources^37,38^. This highlights that pedagogical goals and methods of application may have a greater impact on student learning outcomes than the inherent characteristics of the teaching method.

Our study results revealed that in Human Anatomy and Neuroanatomy laboratories, the academic performance of Q1 in the virtual group was comparable to or even superior to that of the donor group. This suggests that virtual dissection methods are effective in facilitating student learning and comprehension of anatomical structures. It also demonstrates the efficacy of innovative educational technologies in anatomical education. The utility of virtual dissection might extend beyond medical schools where access to hands-on donor dissections may be limited. It can be highly beneficial in non-medical schools, particularly nursing, health sciences, and clinical pathology schools, where opportunities for actual dissection are scarce^39,40^. Therefore, we infer that virtual dissection holds promise as a viable alternative to traditional donor dissection methods.

This study provides novel approach by comparing academic performance of virtual and donor groups in three donor dissection situations: heart extraction, dissection, and observation. In most previous RCT studies in virtual anatomy education, all participants assigned to the donor dissection group conducted the same situation^41^. By varying the donor dissection situations, we can infer that virtual dissection methods are most effective in situations where the heart is only being observed. Consequently, this study provides insight into under what situations virtual dissection may be more appropriate and beneficial than donor dissection.

The findings of this study provide unique evidence that observing a damaged heart during dissection is more challenging in studying anatomical structures compared to instances the heart was extracted or dissected. We speculate that when the heart is damaged during extraction or dissection, specific parts may be lost or altered, leading to difficulties for students in understanding the precise anatomical structures. This observation aligns with previous studies that a damaged or cut structure cannot be reconstructed, making it difficult for the students to revisit a completed dissection on a donor^39,42,43^. Additionally, we assume that the damaged structure compared to their actual anatomical structure might lead students to perceive incorrect information. Therefore, virtual dissection, which allows enhanced learning by freely manipulating, attaching, detaching, and manipulating anatomical structures, may be a promising solution to address these challenges in anatomy education.

One of the key findings of our study was that there was no significant difference in the Q2 score between the donor and virtual groups following the completion of the Human Anatomy and Neuroanatomy laboratories. This may be attributed to the learning effect after the cross-over. This indicates that students from both groups achieved comparable levels of proficiency in Q2 following the completion of these laboratories. In addition, there was no difference in achievement level according to donor and order of exposure to virtual dissection. This suggests that the sequence of exposure may not significantly impact students' performance. Overall, these results imply that both methods can be utilized interchangeably and effectively to achieve similar levels of proficiency in anatomy education.

The study found that HMD, life-size touchscreens, and tablet showed higher student satisfaction than donor across various categories. HMD showed higher satisfaction in understanding of concept and immersion compared to the donor. Labels on virtual anatomical structures likely facilitated effective perception and comprehension. Students would have experienced a more engaging and immersive exploration of anatomical structures during the process of freely investigating them. The life-size touchscreen provided higher satisfaction levels than the donor in terms of esthetics, understanding of the concept, and spatial ability. This suggests that its visually appealing user interface and design contributed to greater satisfaction, aligning with previous studies^44,45^. Clear labeling and multi-angle observation likely enhanced students' understanding of anatomical structures. Accurate labeling is known to enhance learners' understanding and recognition of anatomical components^46,47^. Additionally, the ability to view anatomical structures from various angles contributes significantly to comprehending anatomical structures^48,49^. We speculate that a life-sized touchscreen with a larger display area enhanced visibility, making observation and understanding more convenient. The tablet resulted in superior satisfaction compared to donor in terms of esthetics, understanding of the concept, spatial ability, and continuous use intention. In particular, the aesthetic aspects of neuroanatomy are likely to have higher satisfaction due to its ability to depict color-coded structures and maintain their integrity. Skilled dissection expertise may be necessary for comparable quality in traditional brain dissection. Findings on the continuous use intention support the results that tablets positively impact sustained engagement and learning of anatomy^50^.

The results of this study found that satisfaction with donors was significantly higher in certain categories in Human Anatomy and Neuroanatomy laboratories. Specifically, donors showed higher satisfaction than virtual devices in terms of vividness, anatomical structure, environment and real world. Previous studies have shown that creating 3D virtual models resembling real donors is a major challenge^8,51^. Therefore, researchers have attempted to supplement anatomical knowledge by adding details and textures to 3D virtual models^52^. Numerous studies have focused on developing and validating 3D atlases^53,54^. This suggests that while virtual anatomy content has made significant advancements, it still has limitations in depicting the detail of real humans, which impacts student satisfaction in educational settings.

### Limitations

Our study had several limitations to consider. First, due to limited availability of virtual anatomy devices, future research should be conducted in environments with adequate resources. Second, only two anatomical regions were investigated in this study. investigating only two anatomical regions may limit generalization; expanding to include others would be valuable. Third, the participants were restricted to first-year medical students. Whether these findings can be generalized to different grades must be determined. Fourth, we focused solely on short-term learning outcomes, necessitating further investigation into long-term retention in future studies. Fifth, future studies should compare single virtual learning modalities to donor dissection to better isolate effects. In addition, with a considerable number of participants (n = 108) having prior exposure to VR software, there is a possibility that their familiarity could have influenced their performance and perceptions, potentially favoring the virtual learning format. Finally, due to COVID-19 constraints, the lack of pre-test limits the direct measurement of changes resulting from the educational intervention. While the RCT design measures educational effects, future studies with a pre-test will assess the impact better.

## Conclusion

This study aimed to develop and compare scenario-based virtual dissections with traditional donor dissections in medical education. Virtual dissection yielded superior or equal academic achievement and student satisfaction and can be an effective alternative or supplement to donor dissection, particularly in specific contexts. Furthermore, the satisfaction scores differed in the virtual group. HMD was preferred for understanding of concept and immersion, the life-sized touchscreen received higher scores for esthetics, understanding of the concept, and spatial ability, and tablets were favored for esthetics, understanding of the concept, spatial ability, and continuous use intention. The study demonstrates that virtual dissection can offer valuable learning experiences, improve academic performance, and enhance student satisfaction by providing scenario-based virtual content and incorporating advanced technological devices in medical education. Further research and implementation are necessary to fully explore the benefits of virtual dissection in medical curricula and optimize its integration into medical education programs.

### Supplementary Information

Supplementary Information 1.

Supplementary Information 2.

Supplementary Information 3.

Supplementary Video 1.

Supplementary Video 2.

Supplementary Video 3.

Supplementary Video 4.

Supplementary Information 4.

## Data Availability

The datasets generated during and/or analysed during the current study are not publicly available due to ethical concerns. Data are available from the corresponding author for researchers who meet the criteria for access to confidential data.
