# Supplementary material for: Effectiveness and satisfaction with virtual and donor dissections: A randomized controlled trial
Source: Sci Rep. 2024 Jul 16;14:16388. doi: 10.1038/s41598-024-66292-7 (PMC11252307; doi:10.1038/s41598-024-66292-7)
Supplement: Supplementary file 1 — Supplementary Information 1. [file 41598_2024_66292_MOESM1_ESM.docx]

**Appendix S1.** Content scenarios of “Human Anatomy” laboratory

**Coronary Arteries**

• Let us examine the coronary arteries of the heart.

1) Coronary arteries supply blood to the heart muscle and encircle its exterior.

2) The primary coronary arteries are the left main coronary artery (LMCA) and right main coronary artery. However, in most cases, the LMCA is divided into two branches. The left anterior descending artery, a branch of the left coronary artery, provides blood to the anterior surface of the left side of the heart. The circumflex artery originating from the left coronary artery wraps around the heart muscle and supplies blood to the exterior and posterior regions.

3) In contrast, the right coronary artery (RCA) divides into smaller branches, including the right posterior descending artery and the acute marginal artery. The RCA supplies blood to the right ventricle, right atrium, sinoatrial (SA), and atrioventricular (AV) nodes, which regulate the cardiac rhythm.

4) RCA and the left anterior descending artery supply blood to the heart's middle or septum.

**Chambers of the Heart**

• Let us understand the overall shape of the heart from the front.

1) The upper chambers, called the right and left atria, receive incoming blood. The lower chambers and the right and left ventricles are responsible for pumping blood out of the heart.

2) The heart has a trapezoidal shape with a slightly pointed apex. The sharpness is due to the pointed shapes of both ventricles, which form the apex.

3) Both atria are located towards the base of the heart.

4) Let us observe the right and left atria and ventricles. The two upper chambers are the right and left atria. The two lower chambers are referred to as the right and left ventricles.

**Valves and Cusps of the Heart**

• Let us examine the valves and cusps of the heart.

1) Take a look at the aortic valve comprising three cusps: posterior, right, and left. These cusps were opened to allow blood to flow from the left ventricle to the aorta.

2) Next, observe the pulmonary valve. This valve also has three cusps: anterior, right, and left. They enable blood to be pumped from the right ventricle to the pulmonary artery.

3) Focusing on Tricuspid valve. This valve has three cusps: septal, anterior, and posterior. They enable blood flow from the right atrium to the right ventricle.

4) Finally, let us examine the mitral valve with two cusps: anterior and posterior. This valve allows blood to pass from the left atrium to the left ventricle and prevents backflow from the left ventricle to the left atrium.

• Let us explore the papillary muscles responsible for opening and closing the cusps of the heart.

1. The papillary muscles of the heart are pillar-like muscles located within the ventricular cavities that are connected to their walls.

2) Observe the presence of septal, anterior, and posterior papillary muscles in the right ventricle.

3) Next, observe the anterior and posterior papillary muscles in the left ventricle.

**Conducting System of the Heart**

• Let us explore the conducting system of the heart.

1) The heart needs to maintain synchronization between the atria, causing them to contract together, and then synchronize the ventricles to contract together.

2) The electrical signal responsible for this synchronization begins at the sinoatrial (SA) node and generates a signal. Electrical impulses travel from the SA to the atrioventricular (AV) node. At the AV node, there is a brief delay before the impulses continue along the conduction pathway. They then pass through the bundle of His, dividing them into the right and left bundle branches to stimulate the right and left ventricles, respectively.

**Appendix S2.** Content Scenarios of “Neuroanatomy” laboratory

**Structure inside the Cerebrum**

1) The diencephalon is a complex collection of neural structures located between the cerebrum and midbrain, primarily comprising the thalamus and hypothalamus. The transparent skull allows better visualization of the internal structures. Rotate the structure to observe the positional relationship between the different structures.

2) The upcoming practice involves studying the layers and lobes of the brain, ranging from the ventricles inside to the structures outside.

**Structure of the Ventricle**

1) Let us learn about the third ventricle, located in the innermost part of the brain, and the various ventricles connected to this space.

2) The interventricular foramen connects to the third ventricle, allowing passage from the lateral ventricle in the telencephalon through the aqueduct of the midbrain to the fourth ventricle.

3) Click on the labels of the structures of the lateral ventricle, starting from the front horn to the occipital horn, to observe their locations.

4) Finally, observe the remaining lateral aperture of the fourth ventricle and the central canal of the cord.

**Structure of the Thalamus**

1) Next, let us observe the nuclear structure of the thalamus, located on both sides of the third ventricle.

2) The thalamus, the main mass of the brain, is an egg-shaped structure approximately 4 cm long. It acts as an intermediary between sensory and motor signals and consists of several nuclei.

3) The right part of the thalamus is opaque, whereas the left part is transparent, enabling observation of the external structure and sagittal nucleus.

4) Click on the labels of the medial nuclei of the thalamus located on the inner side of the table to learn their locations and names. Attention should be paid to the enlarged pulvinar at the back end of the thalamus and the open sagittal sulcus on both sides.

5) Finally, observe the pineal body and gland located on the median plane above the superior colliculus.

**Anatomical Relationship between Ventricle, Globus Pallidus, and Striatum**

1) Now, let us examine the relationship between the lateral globus pallidus, medial globus pallidus, and the striatum, located further outside.

2) The striatum can be divided into the caudate nucleus and putamen.

**Anatomical Relationship between Thalamus, Ventricle, Globus Pallidus, and Striatum**

1) Let us review the structures of the thalamus, ventricle, globus pallidus, and striatum.

2) In the following observation, we will take a closer look at the fornix.

**Hypothalamus**

1) Before observing the hypothalamus, let us examine the structure of the fornix.

2) The fornix originates from the hippocampus, forming the crus of the fornix, which ascends and connects to the front of the cerebrum.

3) The crus of the fornix merges in the median plane, forming the body of the fornix, which then becomes the column of the fornix before extending towards the mammillary body.

4) The hypothalamus is bordered by various surface structures. It consists of a mammillary body located behind the hypothalamic boundary, an optic chiasm in the front, and an optic tract on the side.

5) The mammillary body is observed as a pair of structures that rise in a circle just above the midbrain.

6) The optic chiasm meets both optic nerves in the middle plane and the optic tract extends behind the optic chiasm to connect to the lateral geniculate body.

**Cerebral Lobes (Medial Surface)**

1) Observe the frontal lobe, parietal lobe, and occipital lobe on the medial surface of the cerebral hemispheres.

2) Additionally, observe the central sulcus and parietooccipirtal sulcus.

**Front View of the Cerebral Hemisphere**

1) The lamina terminalis forms the front wall of the middle portion. It appears as a thin band. We observed the anterior commissure on the dorsal side of the structure.

2) The septum pellucidum is a thinly illuminated structure located under the corpus callosum that constitutes the inner wall of the lateral ventricle. There is a space between the transparent membranes on the left and right sides called the cavum septum pellucidum. Take a moment to observe and explore this space.

**Cerebral Lobes (Lateral Surface)**

1) The cerebrum is divided into two hemispheres: the cortex (gray matter) on the outside and the nucleus (white matter) on the inside. The cortex is further divided into four lobes: frontal, parietal, temporal, and occipital.

2) Let us observe the frontal, parietal, occipital, and temporal lobes.

**Cerebral Lobes (Lateral Surface** – **Gyrus and Sulcus)**

1) Brain culci and gyri are important anatomical features of the cerebral cortex.

2) Let us observe the lateral sulcus, central sulcus, and parietooccipital sulcus. Also, let us observe the precentral gyrus and postcentral gyrus.

**Top View of the Cerebrum**

1. Now, let us examine the top view of the cerebrum, focusing on the frontal, parietal, and occipital lobes.
2. Next, observe the gyri within the cerebrum. A gyrus refers to a ridge-like elevation on the surface of the cerebral cortex.

**Appendix S3.** Satisfaction questionnaire on the subscales for each category.

**Esthetics**

**· Utility:** I thought the content design was well done, making it easier for me to use.

**· Design:** I thought the content was well-designed and harmonious overall.

**· Vividness:** I could feel the content vividly.

**Understanding of Concept**

**· Labeling:** I could learn well because each anatomical structure was labeled.

**· Desired angles:** I was able to observe structures from my preferred angle and learn about connected blood vessels.

**· Comprehension:** I was able to understand the structures during the experience.

**Reality**

**· Anatomical structures:** I felt the heart was real during the experience.

**· Environment:** I felt like I was in the space of the provided content during the experience.

**· Real word:** I thought the world of the content seemed real during the experience.

**Spatial Ability**

**· Facilitated observation:** I was able to make certain structures transparent or hidden, facilitating the observation of deeper layers of structures.

**· Intuitive understanding:** I thought the anatomical structures and locations were clear and intuitive.

**· Spatial perception:** I was able to improve my spatial perception of anatomical structures.

**Immersion**

**· Desire to continue:** I desired to continue laboratory and did not wish to return to my regular schedule.

**· Sense of being in another space:** I felt like I was in a different space, away from reality.

**· Forget about daily life:** I forgot about my daily life for a while during the experience.

**Continuous Usage Intention**

**· Re-experience:** I would like to experience this again in the future

**· Repetition:** I would like to repeat the experience in the future.

**· Experience in different structures:** I would like to experience the head and neck and upper and lower limbs in the future.
