# Supplementary material for: Effectiveness and satisfaction with virtual and donor dissections: A randomized controlled trial
Source: Sci Rep. 2024 Jul 16;14:16388. doi: 10.1038/s41598-024-66292-7 (PMC11252307; doi:10.1038/s41598-024-66292-7)
Supplement: Supplementary file 2 — Supplementary Information 2. [file 41598_2024_66292_MOESM2_ESM.docx]

**
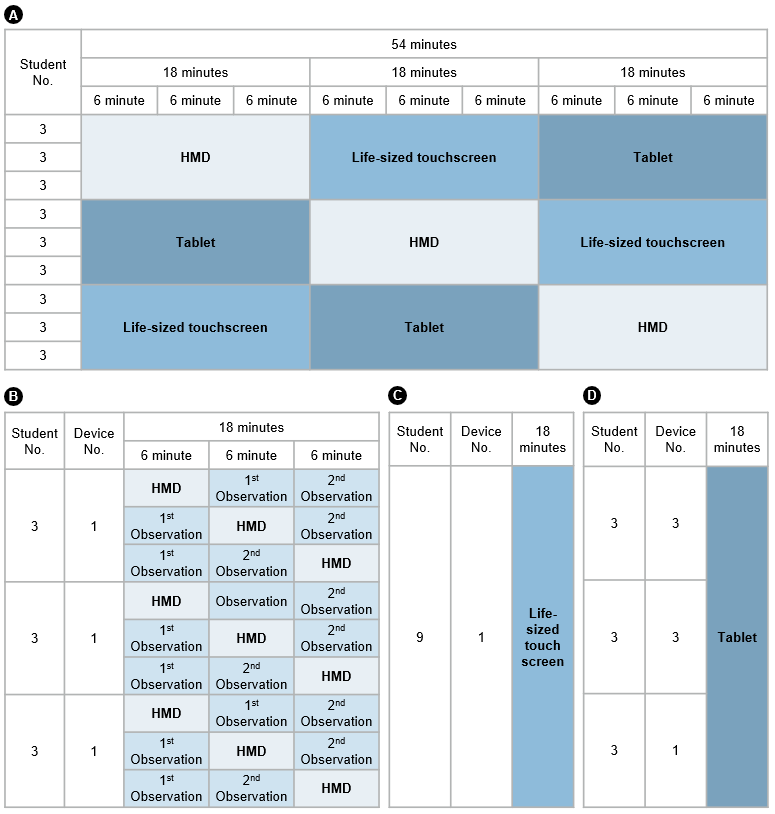
**

**Figure S1.** Time schedule of virtual dissection in the Human Anatomy laboratory using the virtual devices. (A) Head-mounted displays, a life-sized touchscreen, and tablets were used in the laboratory for 54 minutes. (B) Time schedule for head-mounted display-based learning. (C) Time schedule for life-sized touchscrenn-based learning, (D) Time schedule for tablet-based learning. Abbreviation: HMD: head-mounted display.

**
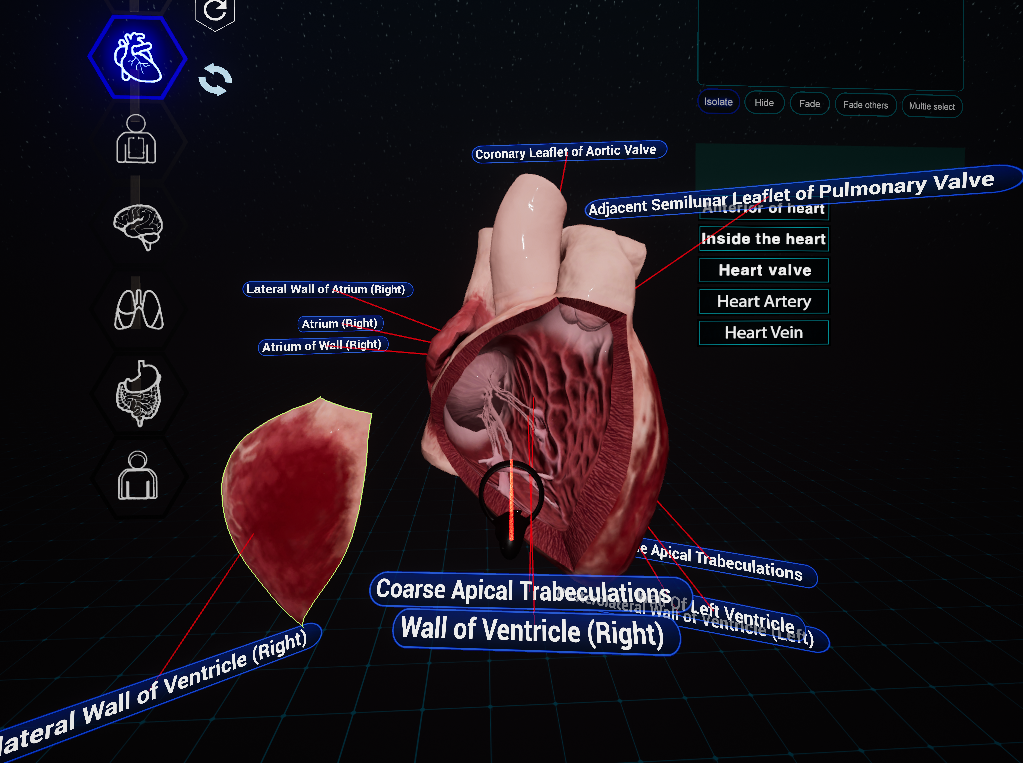
**

**Figure S2.** Heart model content from head-mounted display laboratory. Screenshot of the heart model from the head-mounted display-based laboratory with labels.


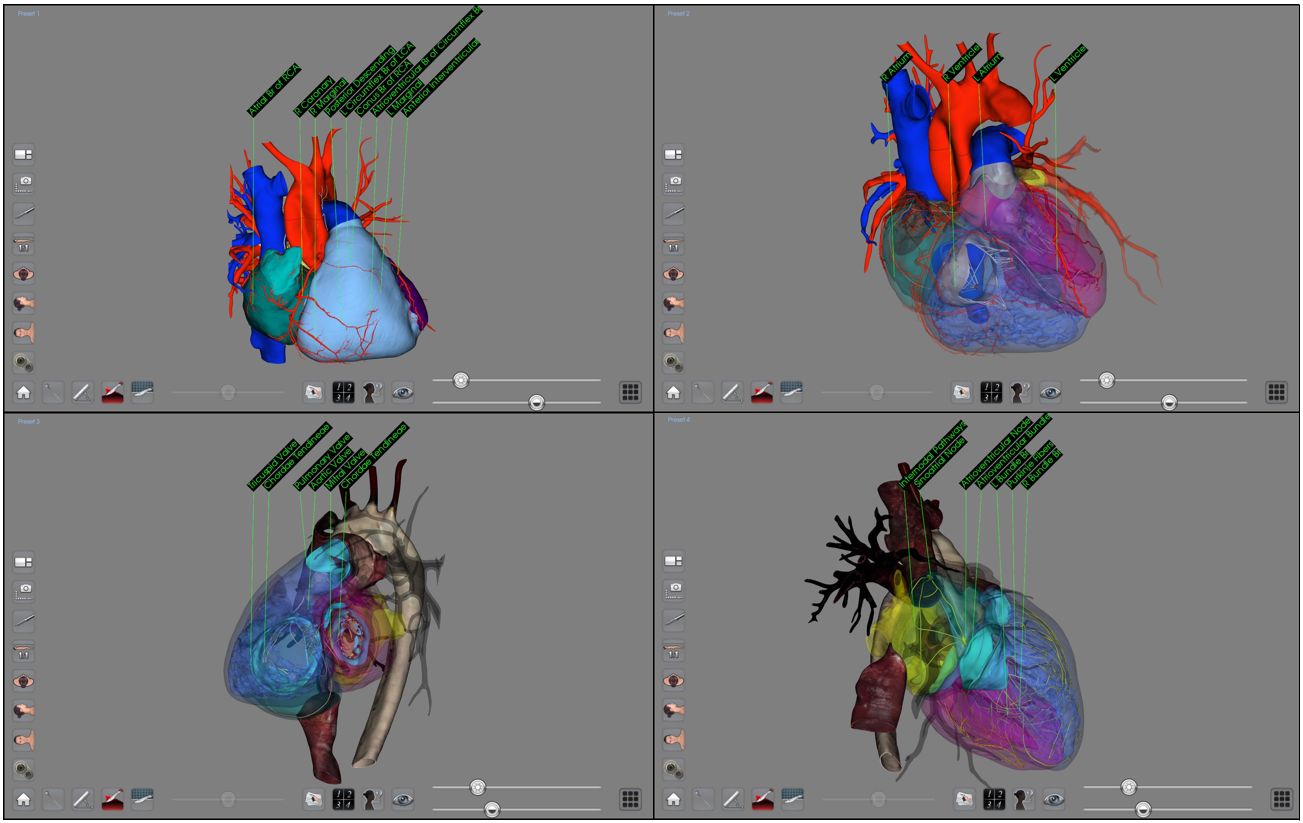


**Figure S3.** Heart model content from life-sized touchscreen laboratory. Screenshots of the heart model from the life-sized touchscreen-based laboratory with labels from various angles.

**
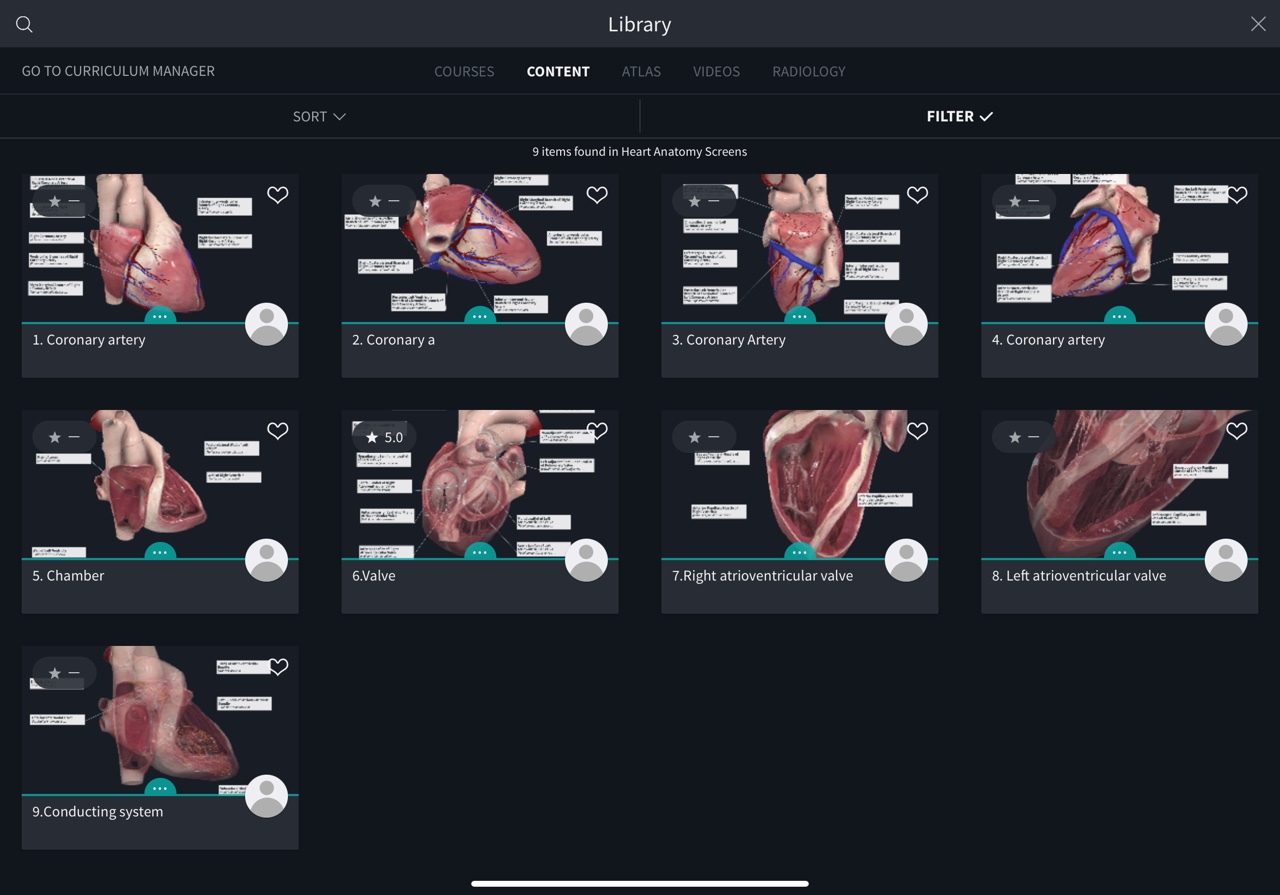
**

**Figure S4.** Heart model content from tablet-based laboratory. Screenshot of the heart models from the tablet-based laboratory with labels from various angles.

**
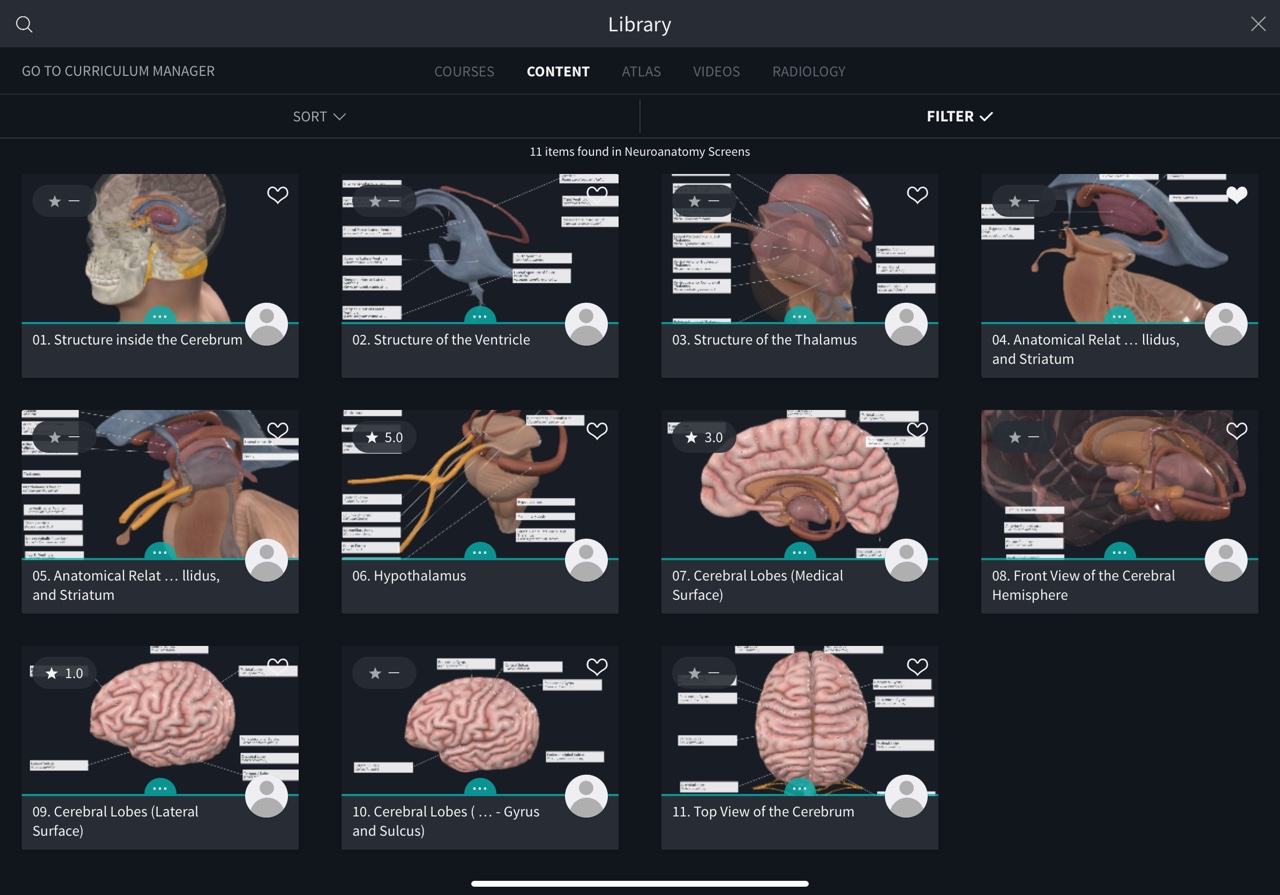
**

**Figure S5.** Diencephalon model content from tablet-based laboratory. Screenshot of diencephalon models from a tablet-based laboratory with labels from various angles.
