# Supplementary material for: Effectiveness and satisfaction with virtual and donor dissections: A randomized controlled trial
Source: Sci Rep. 2024 Jul 16;14:16388. doi: 10.1038/s41598-024-66292-7 (PMC11252307; doi:10.1038/s41598-024-66292-7)
Supplement: Supplementary file 3 — Supplementary Information 3. [file 41598_2024_66292_MOESM3_ESM.docx]

**Table S1.** Basic phase of the AADIE model for the design and development of virtual anatomy education at the SNUCM.

| **ADDIE model** | **Curriculum development process** |
| --- | --- |
|  |  |
| **1) Analysis** | |
| Identify all course design variables, such as learner characteristics, learner’s prior knowledge, and resources available. | Investigate competencies for medical students and anatomical structures difficult for medical students to understand |
| **↓** | |
| **2) Design** | |
| Focus on identifying the course learning goals and how learning materials will be made and designed. | Select and implement digital-based technologies on anatomy teaching. |
| **↓** | |
| **3) Development** | |
| Content creation, including loading of content into a website or LMS. | Make contents scenarios, learning objectives and anatomical 3D images with labeling, and hide and fade functions. |
| **↓** | |
| **4) Implementation** | |
| Install and use instructional instruments and manage instructional materials | Curriculum application |
| **↓** | |
| **5) Evaluation** | |
| Summative evaluation | Analyze curriculum results  (students’ academic performance and satisfaction with the curriculum) |

Abbreviation: ADDIE: analysis, design, development, implementation, and evaluation; SNUCM: Seoul National University College of Medicine; LMS: learning management system
