# Supplementary material for: Effectiveness and satisfaction with virtual and donor dissections: A randomized controlled trial
Source: Sci Rep. 2024 Jul 16;14:16388. doi: 10.1038/s41598-024-66292-7 (PMC11252307; doi:10.1038/s41598-024-66292-7)
Supplement: Supplementary file 8 — Supplementary Information 4. [file 41598_2024_66292_MOESM8_ESM.docx]

**Supplementary video 1.** Heart model content from head-mounted display laboratory.

**Supplementary video 2.** Heart model content from life-sized touchscreen laboratory.

**Supplementary video 3.** Heart model content from tablet-based laboratory.

**Supplementary video 4.** Diencephalon model content from tablet-based laboratory.
